# Supplementary figures and images for: Mechanistic basis for Sgo1-mediated centromere localization and function of the CPC
Source: J Cell Biol. 2022 Jul 1;221(8):e202108156. doi: 10.1083/jcb.202108156 (PMC9253516; doi:10.1083/jcb.202108156)

**B**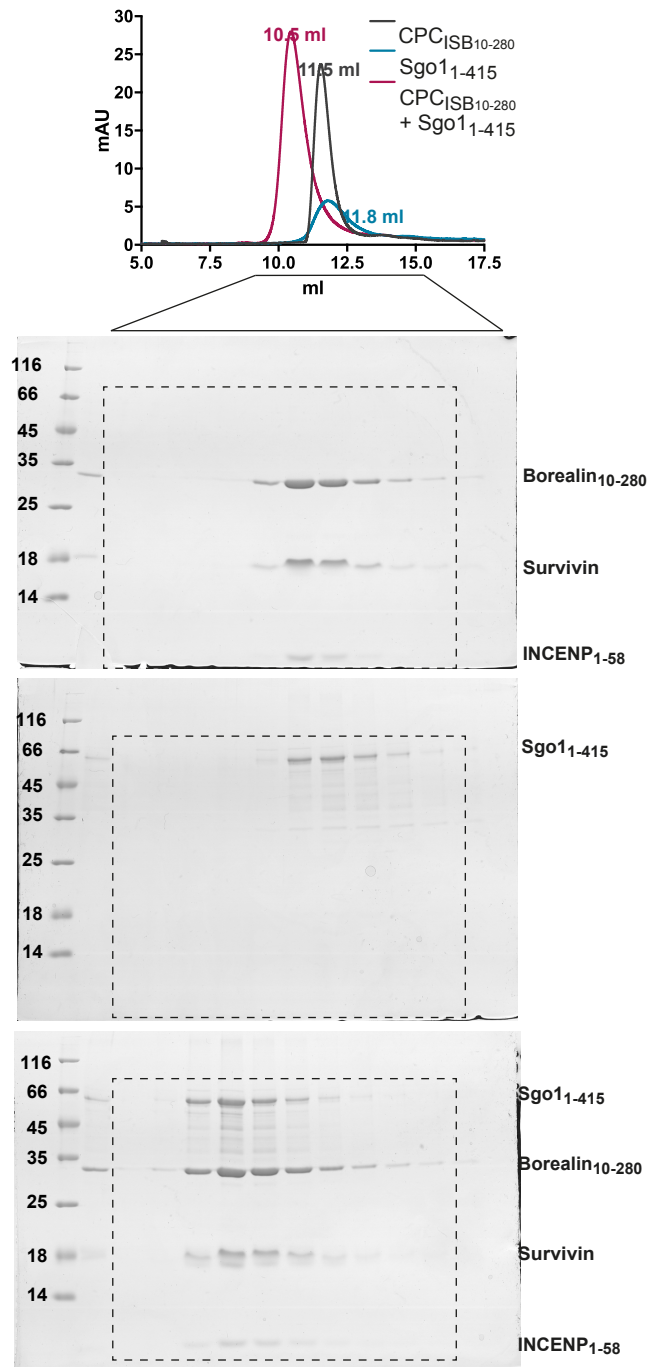

Supplement: SourceData F1 — contains original blots for Fig. 1. [file JCB_202108156_SourceDataF1.pdf]

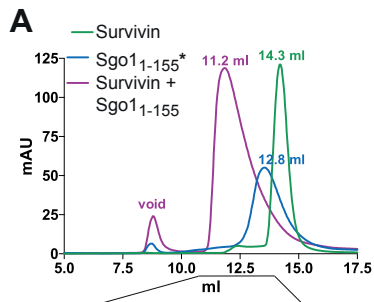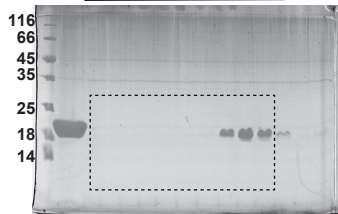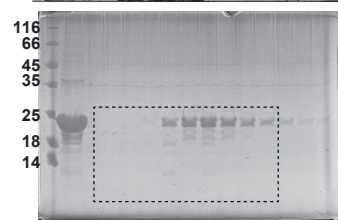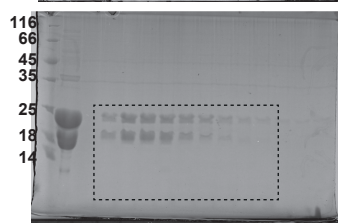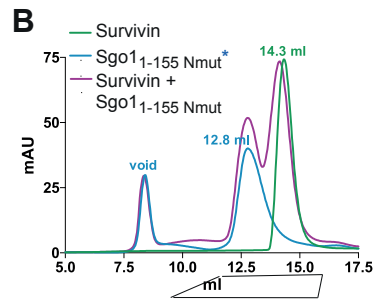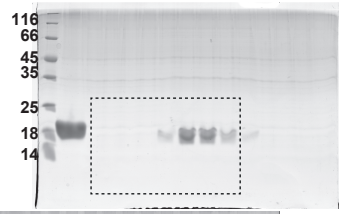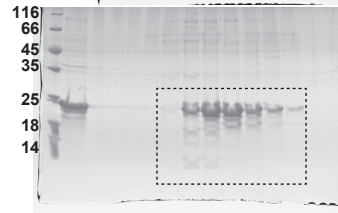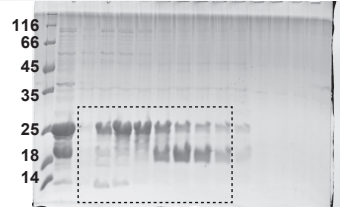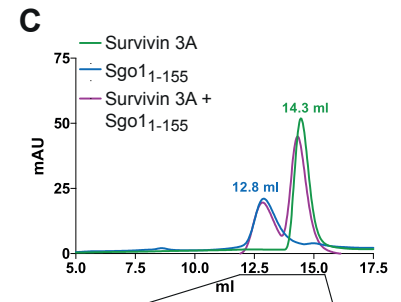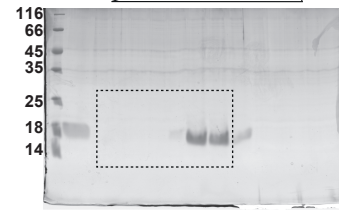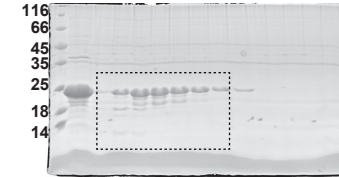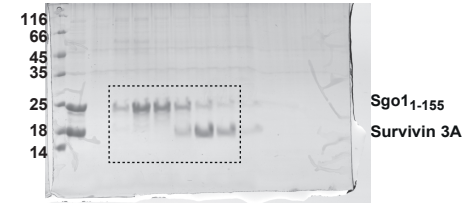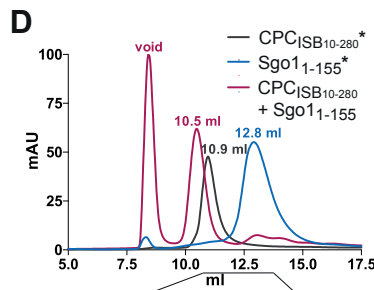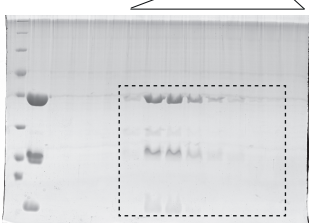

Survivin  
INCENP<sub>1-58</sub>

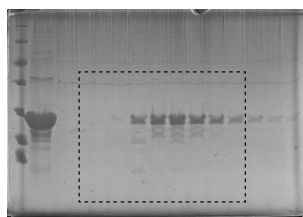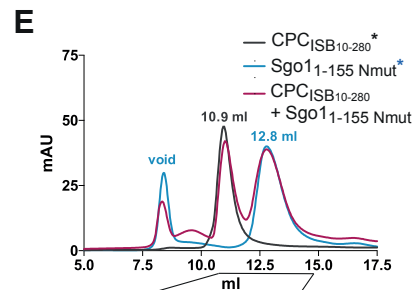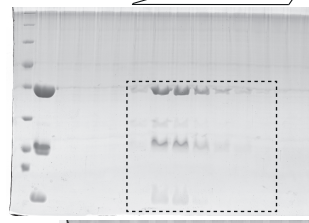

Survivin  
INCENP<sub>1-58</sub>

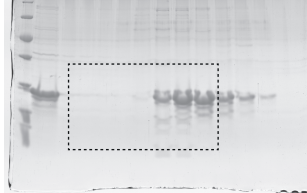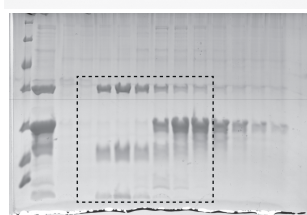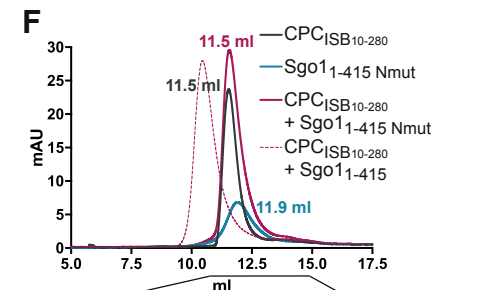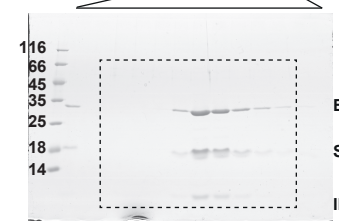

Survivin  
INCENP<sub>1-58</sub>

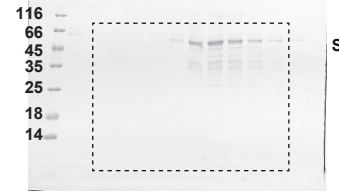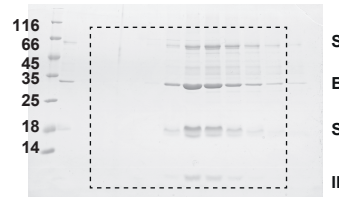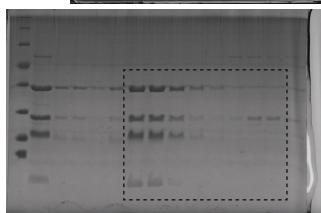

Supplement: SourceData F3 — contains original blots for Fig. 3. [file JCB_202108156_SourceDataF3.pdf]

E

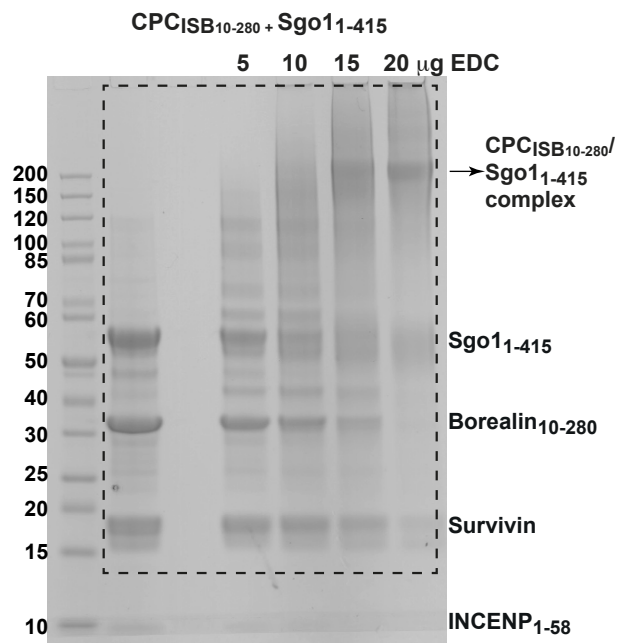

Supplement: SourceData FS1 — contains original blots for Fig. S1. [file JCB_202108156_SourceDataFS1.pdf]

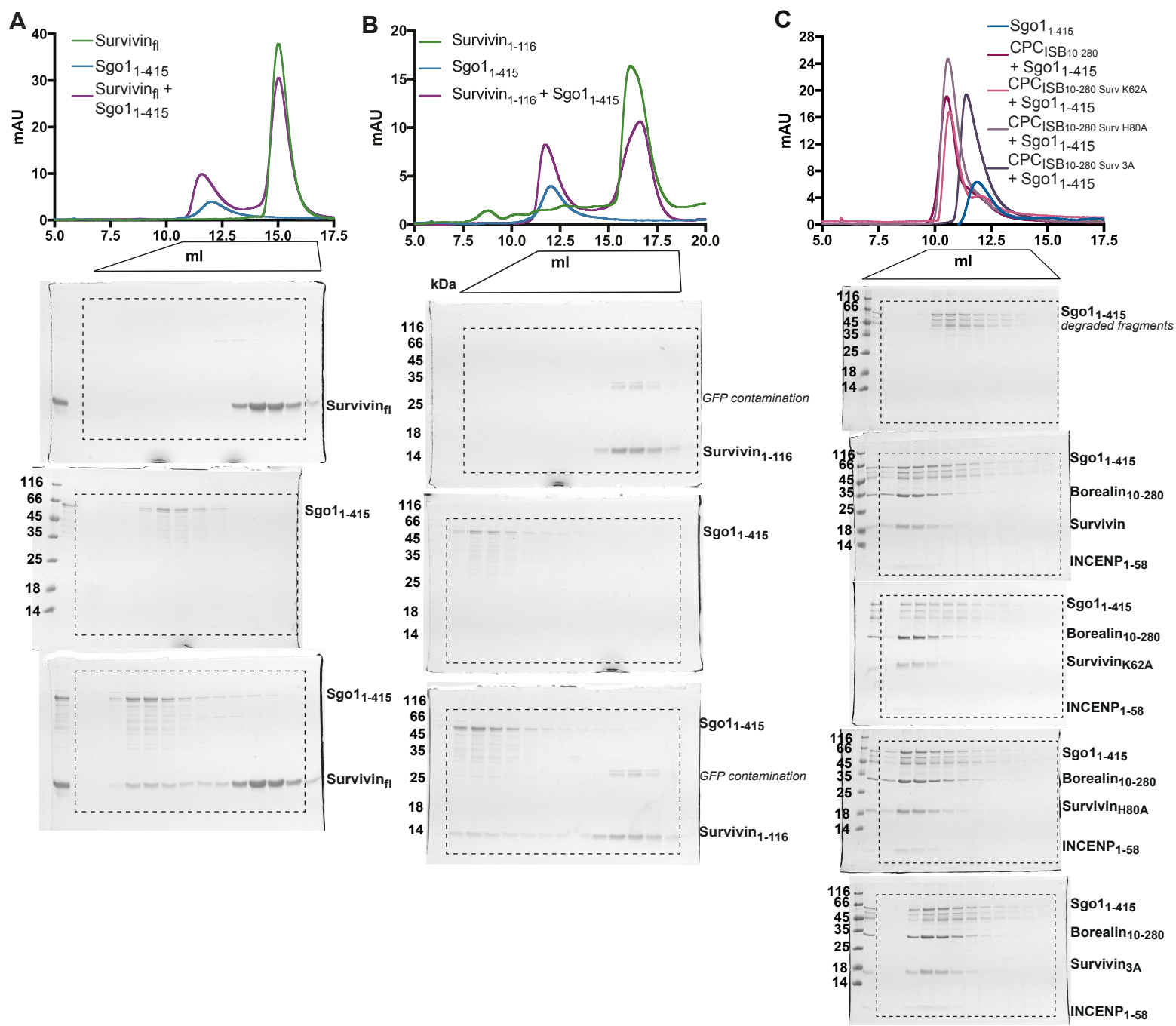

Supplement: SourceData FS2 — contains original blots for Fig. S2. [file JCB_202108156_SourceDataFS2.pdf]

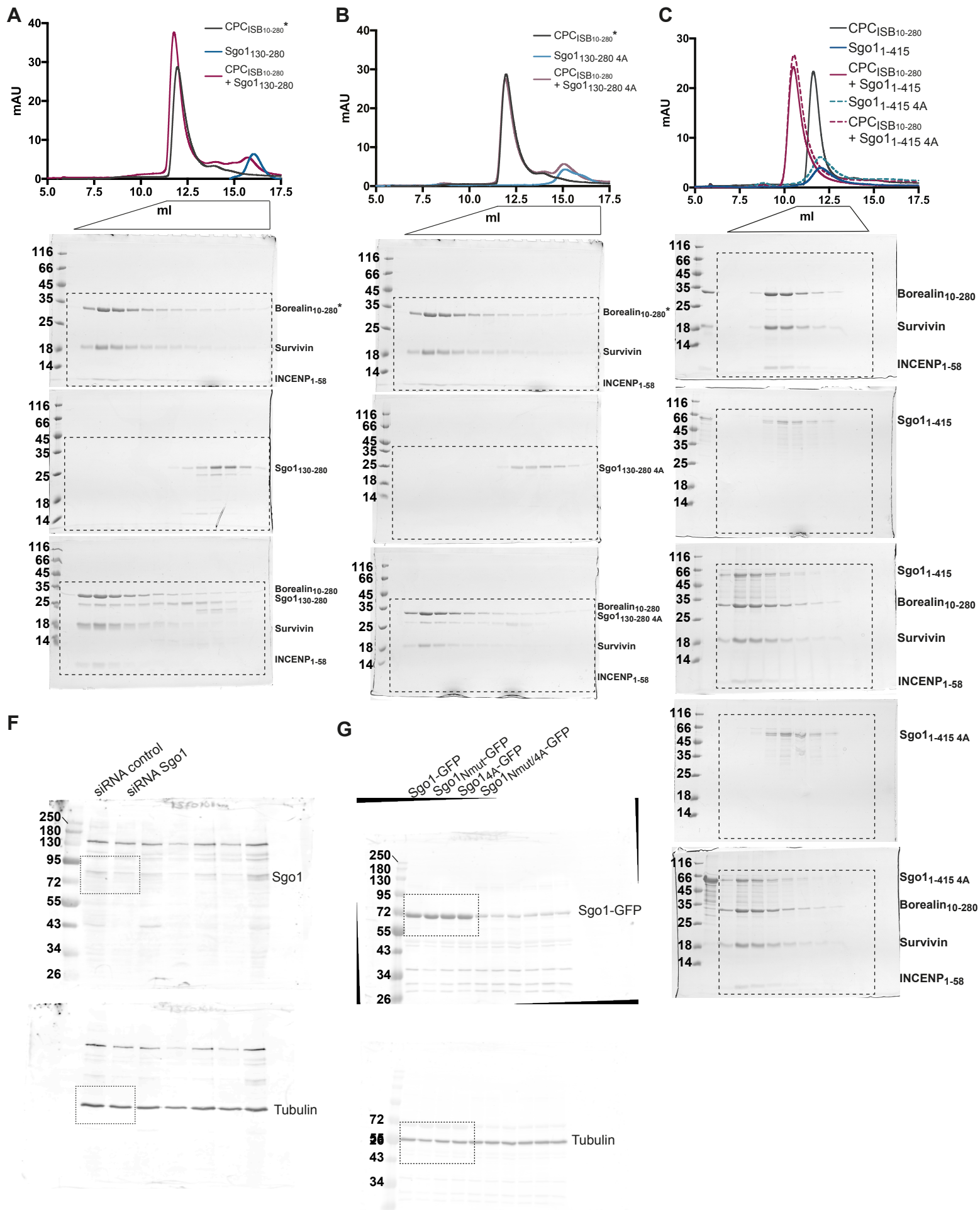

Supplement: SourceData FS4 — contains original blots for Fig. S4. [file JCB_202108156_SourceDataFS4.pdf]
